# Quality by Design-Guided Development of Ketoprofen-Cationic Aspasomes Unlocks the Antifungal Potential of Repurposed Ketoprofen for Topical Therapy

**DOI:** 10.3390/ph19091394

**Published:** 2026-09-02

**Authors:** Moaz A Eltabeeb, Rofida Albash, Mariam Hassan, Sadek Ahmed, Ahmed M. Agiba, Azza A. K. El-Sheikh, Diana E. Aziz

**Affiliations:** 1 Department of Industrial Pharmacy, College of Pharmaceutical Sciences and Drug Manufacturing, Misr University for Science and Technology (MUST), Giza 12585, Egypt; moaz.eltabib@must.edu.eg; 2 Department of Pharmaceutics, College of Pharmaceutical Sciences and Drug Manufacturing, Misr University for Science and Technology (MUST), Giza 12585, Egypt; 3 Department of Microbiology and Immunology, Faculty of Pharmacy, Cairo University, Cairo 11562, Egypt; mariam.hassan@pharma.cu.edu.eg; 4 Department of Microbiology and Immunology, Faculty of Pharmacy, Galala University, New Galala City 43511, Suez, Egypt; 5 Department of Pharmaceutics and Industrial Pharmacy, Faculty of Pharmacy, Cairo University, Cairo 11562, Egypt; sadek.sadek@pharma.cu.edu.eg (S.A.); diana.aziz@pharma.cu.edu.eg (D.E.A.); 6 School of Engineering and Sciences, Tecnológico de Monterrey, Monterrey 64849, Mexico; 7 R&D Directorate, MultiCare Egypt for Pharmaceutical Industries S.A.E., Industrial Area, New Cairo 3, Cairo 11511, Egypt; 8 Basic Health Sciences Department, College of Medicine, Princess Nourah bint Abdulrahman University, P.O. Box 84428, Riyadh 11671, Saudi Arabia

**Keywords:** quality by design, drug repurposing, cutaneous candidiasis, cationic aspasomes, ketoprofen

## Abstract

**Background/Objectives**: Cutaneous fungal infections remain a major therapeutic challenge due to poor skin penetration and emerging antifungal resistance of current treatments. Drug repurposing offers a promising strategy to expand antifungal therapy, with ketoprofen (KPN) recently demonstrating antifungal activity. **Methods**: In this study, KPN-cationic aspasomes (Ca-ASPMs) were developed using a Quality by Design (QbD) approach to enhance topical delivery and maximize the therapeutic potential of repurposed KPN. A D-optimal experimental design was implemented to investigate the influence of ascorbyl palmitate amount (X_1_), ethanol concentration (X_2_), and cationic SAA type (X_3_) on the critical quality attributes of KPN-Ca-ASPMs, namely entrapment efficiency, particle size, and zeta potential. **Results**: Numerical optimization identified an optimum formulation comprising 10 mg ascorbyl palmitate, 5% ethanol, and didodecyldimethylammonium bromide (DDAB) as cationic SAA, with an overall desirability of 0.815. The optimal KPN-Ca-ASPM (F9) exhibited nanosized vesicles (213.24 ± 2.20 nm) and 91.30 ± 10.01% entrapment efficiency. TEM confirmed the spherical morphology of the vesicles, while DSC demonstrated successful incorporation of KPN within the aspasomal matrix. F9 exhibited enhanced in vitro drug release (68% after 6 h) and excellent storage stability. Confocal laser scanning microscopy demonstrated enhanced skin penetration of F9. In a murine cutaneous candidiasis model, F9 significantly enhanced the antifungal efficacy of KPN, producing a 2.989-log reduction in fungal burden compared with the untreated group and significantly outperforming the free drug (*p* = 0.0005). Histopathological examination confirmed restoration of normal skin architecture. **Conclusions**: Collectively, the QbD-guided development of KPN-Ca-ASPM provided a reproducible nanocarrier that significantly enhanced the topical antifungal efficacy of repurposed KPN, highlighting its potential for the treatment of cutaneous candidiasis.

## 1. Introduction

The skin, particularly the epidermis and dermis, is the primary site of cutaneous fungal infections [1]. Among these, cutaneous candidiasis, primarily caused by *Candida albicans*, is the most common fungal skin infection and remains a global health burden [2,3,4]. Although *Candida* species are normal constituents of the skin microbiota, disruption of the cutaneous barrier, occlusive conditions, altered microbial flora, or immunocompromised states can trigger their transition from commensals to pathogenic organisms, leading to cutaneous candidiasis accompanied by pronounced inflammatory responses [5,6]. The growing incidence of antifungal resistance, biofilm-associated persistence, and the limited availability of novel potent antifungal medications have significantly reduced the effectiveness of traditional treatments, necessitating the development of novel therapeutic approaches [4].

To address this challenge, drug repurposing has emerged as a strategic shortcut, identifying new clinical applications for established drugs. Because of their proven safety, tolerability, and pharmacokinetic profiles, this approach reduces the development cost, the number of regulatory licenses needed, and the attrition rates associated with traditional drug development [7,8]. Among repurposing candidates, ketoprofen (KPN), traditionally used as a potent non-steroidal anti-inflammatory drug (NSAID), has attracted attention lately due to its previously reported intrinsic antifungal activity against *Candida albicans* [7]. Unlike conventional agents, KPN possesses a multimodal antifungal mechanism; it inhibits the synthesis of prostaglandins essential for biofilm virulence and directly disrupts fungal cell membrane integrity, while simultaneously providing symptomatic relief by suppressing host inflammation [9]. This combination provides a particular rationale for its investigation in cutaneous candidiasis, where fungal infection is associated with local inflammatory responses.

Despite its therapeutic promise, the successful topical application of KPN remains challenging. Owing to its poor aqueous solubility and high lipophilicity, KPN exhibits limited diffusion through the highly organized stratum corneum and tends to accumulate within superficial skin layers rather than reaching deeper fungal reservoirs. Furthermore, conventional topical dosage forms, e.g., creams and gels commonly used for local treatment of skin fungal infections, show limited therapeutic efficacy against invasive fungal infections due to their poor penetration and drug accumulation in the stratum corneum [10]. Hence, these formulations necessitate high doses with frequent applications to achieve therapeutic levels, which lowers patient adherence and raises the possibility of systemic and local toxicity [4,7]. Therefore, an efficient delivery platform capable of improving skin penetration while maintaining localized drug retention is essential to fully exploit the antifungal potential of repurposed KPN.

Nanotechnology offers a transformative solution to these limitations by enhancing drug retention and providing a controlled drug release that maximizes local therapeutic effects while minimizing systemic exposure [8]. Among lipid-based nanocarriers developed for topical drug delivery, aspasomes have recently attracted increasing attention because they integrate the structural advantages of phospholipid vesicles with the antioxidant and skin-protective properties of ascorbyl palmitate. Being a lipophilic derivative of ascorbic acid, ascorbyl palmitate can contribute to vesicular stability and enhance interaction with skin lipids, facilitating improved cutaneous deposition of encapsulated drugs [11,12]. Consequently, aspasomes represent a promising nanoplatform for overcoming the permeability limitations of poorly soluble therapeutic agents while providing enhanced protection of the encapsulated drugs.

Beyond vesicle composition, surface charge is increasingly recognized as a critical determinant of topical nanocarrier performance. Because both the stratum corneum and fungal cell surfaces have an overall negative charge, imparting a positive surface charge to vesicles can promote electrostatic interactions with the skin and fungal cells, thereby improving vesicle adhesion, extending residence time, and enabling localized drug delivery [13,14]. Moreover, cationic nanovesicles have been reported to promote drug permeation to deeper skin layers through intimate interaction with epidermal lipids while consequently increasing antimicrobial activity by promoting close contact with microbial cell membranes [15]. Hasanovic, A. et al., utilized cationic polymers in formulating minoxidil loaded liposomes, which showed increased stability and increased drug permeation across the skin due to the strong interaction between the positively charged vesicles and the negative skin surface [16]. Consequently, the vesicles go deeper into the skin, distracting the tight junctions of the lower epidermal layers. Accordingly, engineering cationic aspasomes may provide a dual-targeting strategy capable of enhancing both skin localization and antifungal performance of repurposed KPN.

Although numerous vesicular systems have been explored for topical drug delivery, their formulation was frequently developed through a traditional approach using one variable at a time (OVAT) that inadequately captures the complex interactions among the formulation variables. In addition, being time consuming, OVAT frequently results in sub-optimal formulation with poor reproducibility and limited robustness [17]. To overcome these limitations, Quality by Design (QbD) has emerged as a systematic risk-based approach that evaluates the formulation and manufacturing process in order to develop a controlled process that guarantees the product’s quality [18]. Identifying critical material attributes (CMAs) and critical process parameters (CPPs) in QbD is essential for preserving critical quality attributes (CQAs) and guaranteeing a systematic approach to pharmaceutical development. Within the QbD framework, Design of Experiments (DoE) plays an important role in understanding the formulation CMAs and process variables (CPPs) through simultaneous evaluation of multiple variables, their interactions, and evaluation of their effects on CQAs [19]. Hence, integrating a QbD approach and DoE in nanoparticle formulations could significantly save time and cost, minimize trials and errors, and consequently produce more predictable outcomes with minimal need for frequent development changes [20].

Therefore, the present study aimed to rationally engineer ketoprofen cationic aspasomes (KPN-Ca-ASPMs) as a novel topical nanoplatform for the treatment of cutaneous candidiasis using a QbD approach. A D-optimal experimental design was employed to investigate the effects of ascorbyl palmitate amount (X_1_), ethanol concentration (X_2_), and cationic SAA type on the CQAs, including entrapment efficiency, particle size, and zeta potential, enabling the identification of the optimal formulation with predefined quality attributes. The optimal KPN-Ca-ASPMs were comprehensively characterized and evaluated for physicochemical stability, drug release, skin penetration, and antifungal efficacy through in vitro microbiological assays and in vivo assessment in a murine model of cutaneous candidiasis, thereby providing preclinical evidence supporting the successful repurposing of KPN as a topical antifungal therapy.

## 2. Results

### 2.1. Analysis of D-Optimal Design

In alignment with the QbD strategy, the analysis of the D-optimal design results provided a roadmap for controlling the critical quality attributes (CQAs) of the KPN-Ca-ASPMs. The statistical optimization via D-optimal design can shift the emphasis from the traditional optimization process to a risk-based understanding of the formulation’s design space [21]. By employing a D-optimal experimental design, we were able to simultaneously evaluate the impact of two quantitative critical material attributes (CMAs), ascorbyl palmitate amount (X_1_) and ethanol concentration (X_2_), alongside the categorical factor of cationic SAA type (X_3_). This systematic strategy was essential to establish the mathematical relationship between these variables and the CQAs, namely entrapment efficiency (Y_1_: EE%), particle size (Y_2_: PS), and zeta potential (Y_3_: ZP). Model fitting showed EE% was best described by a cubic response model, whereas the PS and ZP followed the 2FI model. The adequacy of the selected models was verified by adequate precision, which possessed values above four for all responses (Table 1), indicating adequate signal-to-noise ratios. It is also notable that the predicted *R*^2^ values were in good harmony with the adjusted *R*^2^ values for all responses, confirming that the selected model fits the data adequately [22].

### 2.2. Analysis of EE%

**Figure 1:**
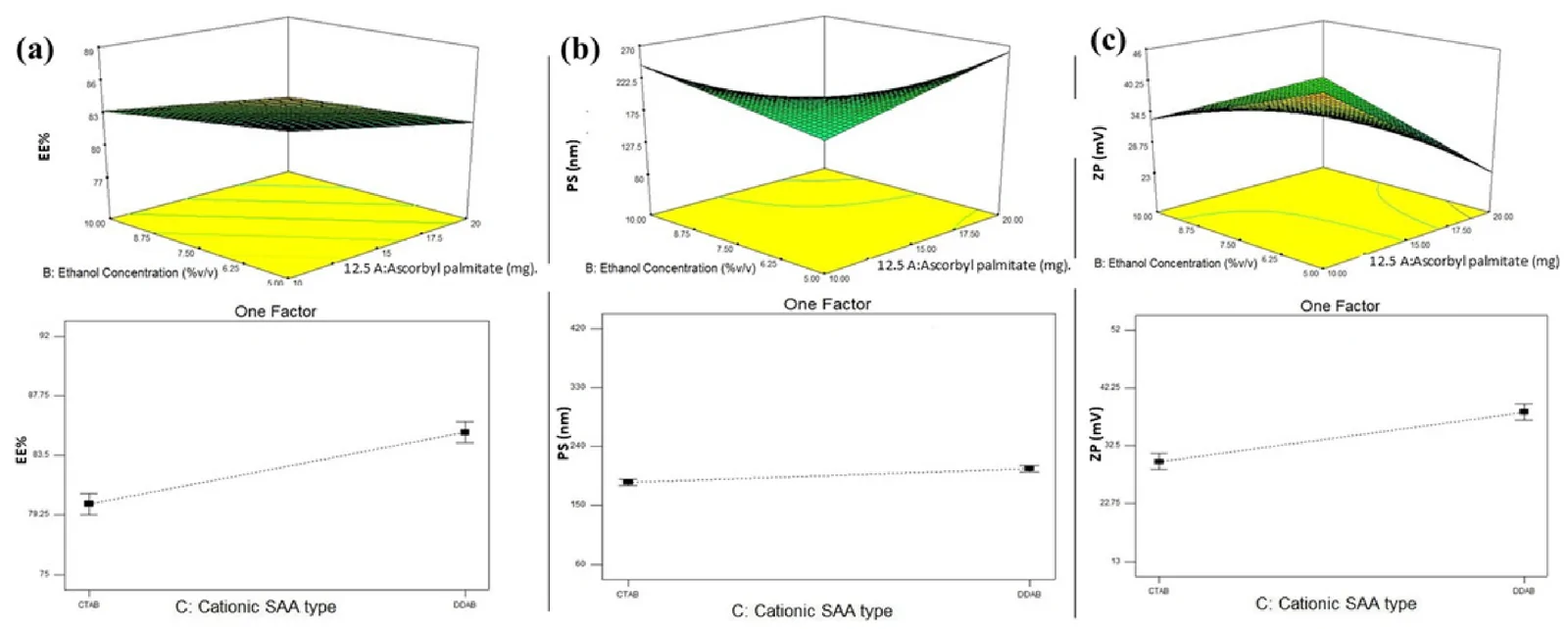
Response surface plots illustrating the combined effects of ascorbyl palmitate amount (A) and ethanol concentration (B) on (**a**) entrapment efficiency (EE%), (**b**) particle size (PS), and (**c**) zeta potential (ZP) of KPN-Ca-ASPMs. The lower panels represent one-factor plots illustrating the effect of cationic SAA type (C) on the corresponding responses.

The EE% is a crucial CQA to ensure that a high payload of KPN was successfully entrapped within the aspasomal core, ensuring sustained antifungal activity [7]. The EE% of the prepared KPN-Ca-ASPMs ranged from 75.00 ± 0.76% to 91.30 ± 10.01% (Table 2). As shown in Figure 1a, statistical analysis (*p* < 0.05) showed that all investigated factors had a significant impact on the EE%. Increasing the ascorbyl palmitate amount (X_1_) from 10 to 20 mg significantly reduced the EE%. When the quantity of ascorbyl palmitate, a surfactant-like lipid, exceeded the optimal threshold, the excessive surfactant boosted the formation of mixed micelles which consequently increased the KPN solubility in the dispersion phase rather than bilayer encapsulation [23,24]. Furthermore, ascorbyl palmitate contributes to the rigidity of the vesicular bilayer. Therefore, an overly high ascorbyl palmitate amount can negatively affect the optimal packing arrangement of the vesicular bilayer, leading to a reduction in the interstitial space available for KPN molecules to intercalate [25]. Considering the ethanol concentration (X_2_), it was shown to have a negative effect on the EE%. It could be related to the fluidizing effect of ethanol on ascorbyl palmitate bilayers, which increases membrane permeability and reduces the ability of the forming vesicles to efficiently retain KPN, thereby decreasing the EE% [26]. On the other hand, at higher ethanol concentration, KPN might favor exerting higher solubility in the hydroethanolic phase rather than partitioning into the lipid bilayer during self-assembly, resulting in less drug entrapment. As shown in Figure 1a, aspasomes prepared using CTAB showed significantly lower EE% compared to DDAC-based vesicles due to the lower lipophilicity of CTAB (log P = 8) compared to DDAB (log P = 11.8). This higher lipophilicity of DDAB promoted the intercalation of lipophilic KPN within the lipophilic bilayer core, resulting in a greater EE% than the aspasomes containing CTAB [27].

### 2.3. Analysis of PS

Preparing aspasomes with a small PS enhances its deep penetration through the skin more than with larger ones, which deposit at the surface of the SC [28]. Hence, PS is a paramount CQA in topical delivery and the primary determinant of the ability of aspasomes to bypass the stratum corneum and penetrate into the deeper epidermal layers. As shown in Table 2, the PS of KPN-Ca-ASPMs ranged from 66.38 ± 9.87 nm to 313.71 ± 2.19 nm. Statistical analysis of the D-optimal design confirmed that all three independent variables (X_1_, X_2_, and X_3_) acted as critical process and material attributes, exerting a significant modulatory effect on the aspasomal PS (*p* < 0.05).

As shown in Figure 1b, increasing ascorbyl palmitate amount (X_1_) resulted in the formation of aspasomes with smaller PS. The surface activity of ascorbyl palmitate (HLB = 8.4) can be linked to its negative effect on PS. Increasing the ascorbyl palmitate amount lessens the lipid/water interfacial tension, maintains steric stabilization, and enhances surface curvature, resulting in the formation of smaller vesicles [12]. Statistical analysis revealed that ethanol concentration (X_2_) exerted a significant antagonistic effect on the PS of KPN-Ca-ASPMs. This reduction in size may be attributed to an ethanol-induced interpenetrating hydrocarbon chain phase, which physically reduces the thickness of the vesicular membrane [29]. Furthermore, increasing ethanol concentration effectively lowers the main transition temperature of the aspasomal lipid matrix, inducing a state of partial fluidization within the bilayers and the formation of smaller, more kinetically stable nanoparticles during the solvent injection process [30]. CTAB-based aspasomes are smaller than DDAB-based ones. This might be explained by the much greater quantity of KPN trapped in the hydrophobic core of aspasomes, which consequently led to PS expansion [31].

### 2.4. Analysis of PDI

The homogeneity and size distribution of nanoscale vesicles are crucially indicated by the polydispersity index (PDI). For pharmaceutical applications, a monodisperse system with appropriate homogeneity is indicated by PDI values less than 0.5 [32]. As shown in Table 2, the PDI of the formulated KPN-Ca-ASPMs fluctuated from 0.232 ± 0.034 to 0.450 ± 0.003, confirming the relative homogeneity and controlled particle size distribution of the formed vesicles [33]. Additionally, ANOVA showed that none of the formulation variables under investigation had a significant impact on PDI (*p* > 0.05). This finding implies that compositional changes within the examined design space had no effect on the vesicular homogeneity and consequently PDI was excluded from the optimization process.

### 2.5. Analysis of ZP

The surface charge density, expressed by the ZP was monitored as a fundamental indicator of the system’s kinetic stability, providing insight into whether aspasomes will maintain their integrity and remain as a uniform dispersion within the final gel matrix [34]. As shown in Table 2, the developed aspasomes exhibited ZP ranging from 13.40 ± 0.60 to 51.70 ± 0.65 mV. All formulae had positive ZP due to the presence of cationic SAA in their constructs.

The influence of ascorbyl palmitate amount (X_1_), ethanol concentration (X_2_), and type of cationic SAA (X_3_) on the ZP of KPN-Ca-ASPM is graphically illustrated in Figure 1c. The ANOVA results for the ZP model demonstrated that only the ascorbyl palmitate amount (X_1_) and type of cationic SAA (X_3_) had a significant impact on the ZP values of KPN-Ca-ASPMs (*p* ˂ 0.0001). Considering the ascorbyl palmitate amount (X_1_), this antagonistic effect can be attributed to the charge dilution phenomenon, whereby increasing the proportion of the non-ionic lipid relative to the fixed amount of cationic surfactant reduces the surface charge density of the vesicles. Consequently, fewer positively charged surfactant molecules are exposed per unit surface area, leading to lower zeta potential values.

Considering the type of cationic SAA (X_3_), aspasomes prepared with DDAB exhibited significantly higher positive ZP than those containing CTAB. This may be attributed to the greater hydrophobicity and double-chain structure of DDAB, which promote its stable incorporation and orientation within the phospholipid bilayer while exposing its quaternary ammonium head groups at the vesicle surface. In contrast, the single-chain structure of CTAB may result in less efficient anchoring within the lipid membrane, leading to a lower surface charge density.

### 2.6. Optimization of KPN-Ca-ASPMs

The optimization process, performed within the QbD framework using the established desirability function approach, identified F9 as the optimal KPN-Ca-ASPM which achieved the highest overall desirability (0.815) and was prepared using 10 mg ascorbyl palmitate, 5 mL ethanol, and DDAB as cationic SAA. It showed an EE% of 91.30 ± 10.01%, PS of 213.24 ± 2.20 nm, and ZP of 51.70 ± 0.65 mV. The predicted and observed outputs of F9 were compared and the results are shown in Table 3. The high degree of agreement between the predicted and observed values of F9 confirmed the adequacy of the selected QbD model and highlighted its ability to correctly predict the formulation performance. Following its selection as the optimal formulation, F9 was independently prepared several times to further assess its reproducibility before subsequent in vitro and in vivo characterization. The mean EE% value was 91.16 ± 0.89% (*n* = 5), demonstrating substantially lower variability upon repeated preparation. F9 was subsequently selected for further in vitro and in vivo characterization.

### 2.7. In Vitro Testing of the Optimal KPN-Ca-ASPM

#### 2.7.1. In Vitro Drug Release

**Figure 2:**
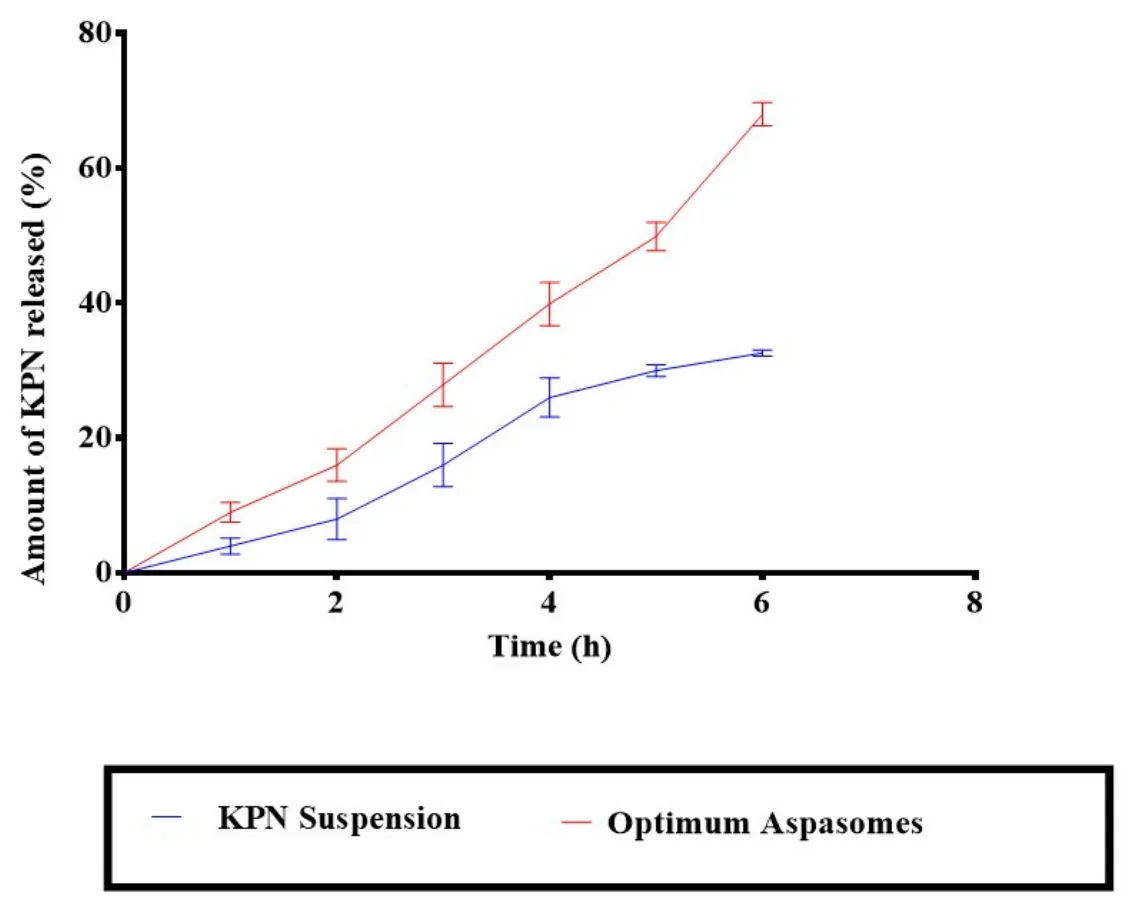
In vitro release profiles from the optimal formula (F9) compared to KPN suspension.

The in vitro release profiles demonstrated a markedly enhanced release of KPN from the optimized aspasomal formulation compared with the drug suspension throughout the study period (Figure 2). After 6 h, aspasomes released approximately 2-fold more drug than the suspension (68% vs. 32.6%). The superior release behavior of the aspasomal formulation may be attributed to the nanosized vesicles, which provide a substantially larger surface area for drug diffusion [35]. Furthermore, the amphiphilic nature of the vesicular components can improve the apparent solubility and partitioning of the poorly water-soluble KPN into the aqueous medium [7]. In contrast, the limited release from the suspension is primarily governed by the poor aqueous solubility and slow dissolution rate of crystalline KPN. To further characterize the release behavior, the release data were fitted to different kinetic models. The KPN suspension showed the best fit to the Hixson–Crowell model (R^2^ = 0.963), whereas the release from F9 was best described by the diffusion model (R^2^ = 1), suggesting that diffusion through the aspasomal matrix was the predominant mechanism governing KPN release from the optimized formulation.

#### 2.7.2. Transmission Electron Microscopy

**Figure 3:**
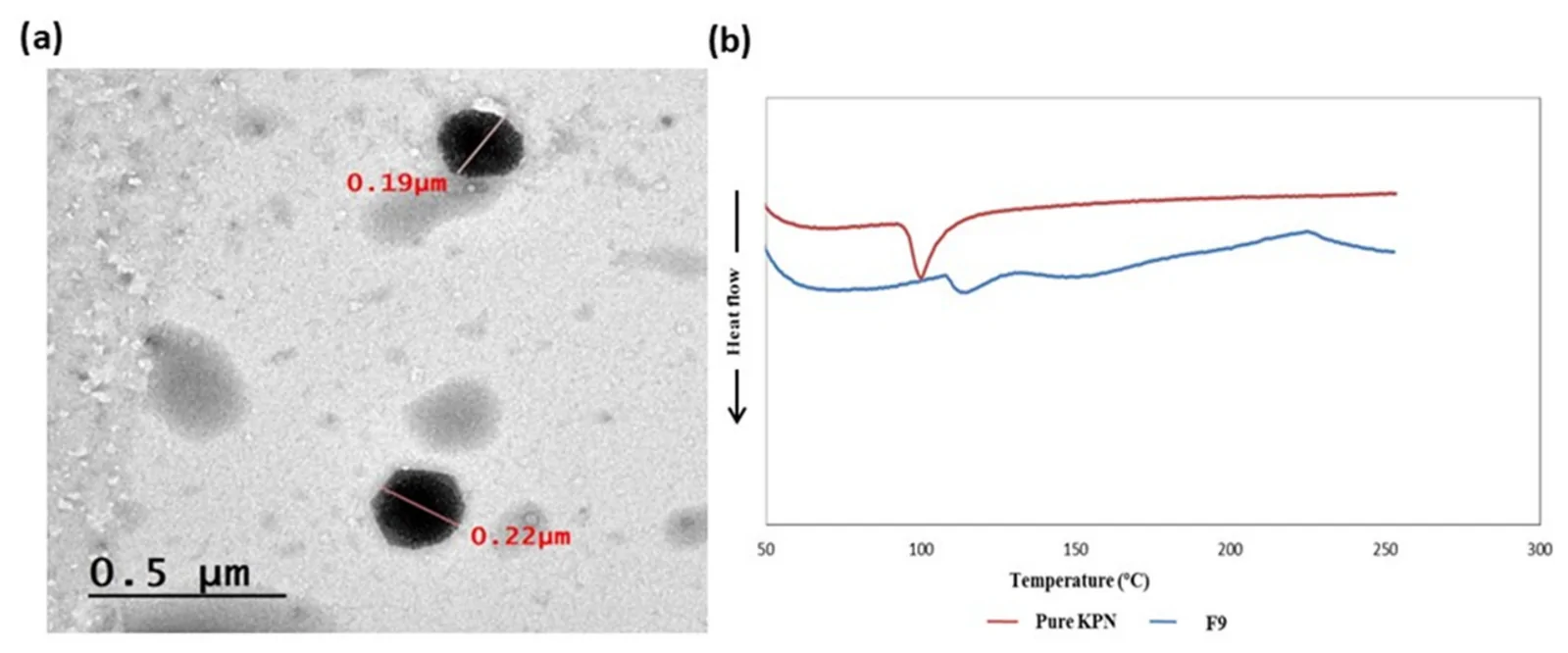
TEM micrograph (**a**) and DSC thermograms (**b**) of the optimal KPN-Ca-ASPMs (F9).

TEM analysis confirmed the successful formation of well-defined, distinct aspasomal vesicles with a predominantly spherical architecture (Figure 3a) [12]. The vesicles showed no signs of aggregation or structural deformation, indicating that the formulation procedure maintained vesicular integrity [7]. The mean vesicle diameter determined from the measurable TEM vesicles was 201 ± 38.36 nm (*n* = 13), which was comparable to the particle size determined by dynamic light scattering (213.24 ± 2.20 nm). However, the two techniques measure particle dimensions under different conditions: DLS determines the hydrodynamic diameter of vesicles dispersed in liquid, including their associated hydration layer, whereas TEM measures the apparent physical diameter of dried vesicles following sample preparation. Therefore, some differences between the TEM and DLS measurements are expected.

#### 2.7.3. Differential Scanning Calorimetry (DSC)

The DSC thermogram of pure KPN exhibited a sharp endothermic melting peak at approximately 99.9 °C, confirming its crystalline nature (Figure 3b) [36]. In contrast, F9 displayed a markedly reduced endothermic peak, along with a minor shift of the residual peak to approximately 114.6 °C. The substantial reduction in the melting enthalpy suggests a marked reduction in the crystallinity of KPN following incorporation into the aspasomal lipid matrix, which may indicate that a considerable fraction of the drug exists in a less crystalline or amorphous state [24]. The slight shift in the residual thermal peak may be attributed to interactions between KPN and the vesicular components, which restricted molecular mobility and consequently increased the thermal energy required for melting. The aforementioned results confirmed the successful drug entrapment within the aspasomal vesicles.

#### 2.7.4. Effect of Short-Term Storage

The physical stability of F9 was evaluated after storage under refrigerated conditions (2–8 °C) for 90 days. Throughout the storage period, F9 exhibited an EE% of 90.00 ± 1.88%, a PS of 384.43 ± 20.54 nm, a PDI of 0.366 ± 0.0453, and a ZP of +48.98 ± 2.12 mV. Statistical comparison between the fresh and stored formulations using Student’s *t*-test revealed no significant differences in the evaluated physicochemical characteristics (*p* > 0.05). From a formulation perspective, the numerical increase in PS may suggest some degree of vesicular aggregation or particle growth during prolonged storage. Nevertheless, the EE%, PDI, and ZP remained relatively consistent after storage, indicating preservation of the overall physicochemical characteristics of the formulation. These findings assure the physicochemical short-term stability of the optimal aspasomes formulation and its resistance to drug leakage under refrigerated storage conditions.

### 2.8. Microbial and In Vivo Analysis

#### 2.8.1. In Vitro Antifungal Activity

With a minimum inhibitory concentration (MIC) of 5 ± 0 mg/mL, KPN showed encouraging antifungal efficacy against *Candida albicans* ATCC10231.

#### 2.8.2. In Vivo Fungal Skin Infection Model

**Figure 4:**
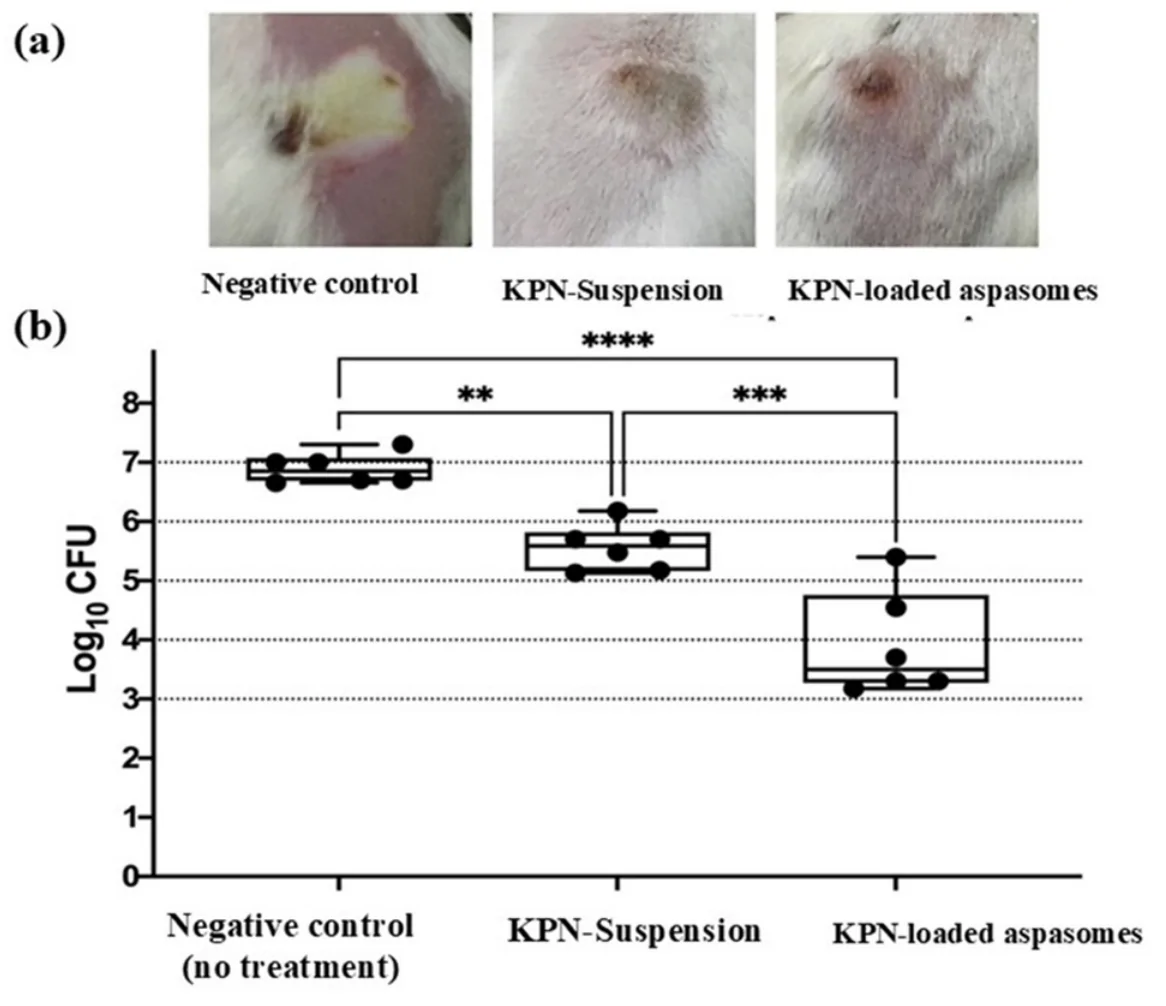
Efficacy of KPN-Suspension and its optimal formula (F9) in an in vivo murine model of fungal skin infection. (**a**) Photo image of the efficacy of different treatment groups on fungal skin infection in the posterior backs of mice at the end of the experiment. (**b**) Efficacy of different treatment groups on the fungal load in murine model fungal skin infection. Each data point in the figure represents a mouse. Results are expressed as mean ± standard error. **, ***, and **** indicate that the difference is significant at *p* < 0.01, 0.001, and 0.0001, respectively, (one-way ANOVA, Tukey’s post hoc test).

The in vivo antifungal activity of the KPN-suspension and F9 was tested against *Candida albicans* ATCC10231 using a murine model of cutaneous candidiasis (Figure 4). Three groups of male BALB/C mice (*n* = 6) were injected intradermally with *Candida albicans* ATCC10231 suspension. At 48 h post-infection, an abscess developed at the site of infection. Visual assessment of the infection sites revealed significant differences in healing between the groups (Figure 4a). The KPN-suspension and F9 groups showed considerable reduction in lesion size and inflammation compared to the negative control group. Quantitative microbiological analysis corroborated these visual findings (Figure 4b). Both free KPN and F9 significantly decreased the fungal count of *Candida albicans* compared to the negative control group (one-way ANOVA, Tukey’s post hoc test, *p* < 0.01) (Figure 4). Crucially, the antifungal activity of F9 was significantly superior to that of the free drug (one-way ANOVA, Tukey’s post hoc test, *p* = 0.0005), supporting the potential of the aspasomal delivery system to enhance the antifungal activity of KPN in this experimental model. The fungal count retrieved from the F9 treated group was 2.989 logs lower than that of the negative control group. The fungal count retrieved from the free KPN treated group was 1.332 logs lower than that of the negative control groups. These findings provide preliminary preclinical evidence that incorporation of KPN into the aspasomal system may enhance its antifungal activity against cutaneous candidiasis. While free KPN exhibited significant intrinsic antifungal activity, likely due to its ability to interfere with fungal metabolic pathways or induce oxidative stress, its efficacy was limited when applied in its free form [7]. In contrast, the encapsulation of KPN into aspasome nanoparticles resulted in a nearly 3-log reduction in fungal recovery. The significantly higher efficacy of the optimal KPN-Ca-ASPMs compared to the free drug can be attributed to the unique properties of aspasomes. In the present study, the enhanced skin distribution of F9 and its improved antifungal efficacy were experimentally demonstrated. These findings suggest that the vesicular system may facilitate improved localization and penetration of KPN within the skin. As vesicular carriers are composed of ascorbyl palmitate, aspasomes act as active delivery systems that combine the skin-penetrating capabilities of vesicles with the antioxidant and anti-inflammatory benefits of Vitamin C derivatives. These vesicles may facilitate deeper drug penetration through the stratum corneum and into the deeper epidermal layers where *Candida albicans* hyphae proliferate. Furthermore, the localized and sustained release provided by the aspasome matrix ensures that KPN remains at the site of infection at therapeutic concentrations for an extended period, ultimately leading to expected superior clinical and microbiological outcomes compared to conventional drug administration. Although the findings obtained in the murine model are promising, translation to human cutaneous candidiasis should be approached cautiously, as differences in skin structure, physiology, and disease characteristics may affect clinical outcomes.

#### 2.8.3. Skin Distribution Observed by Confocal Laser Scanning Microscopy (CLSM)

**Figure 5:**
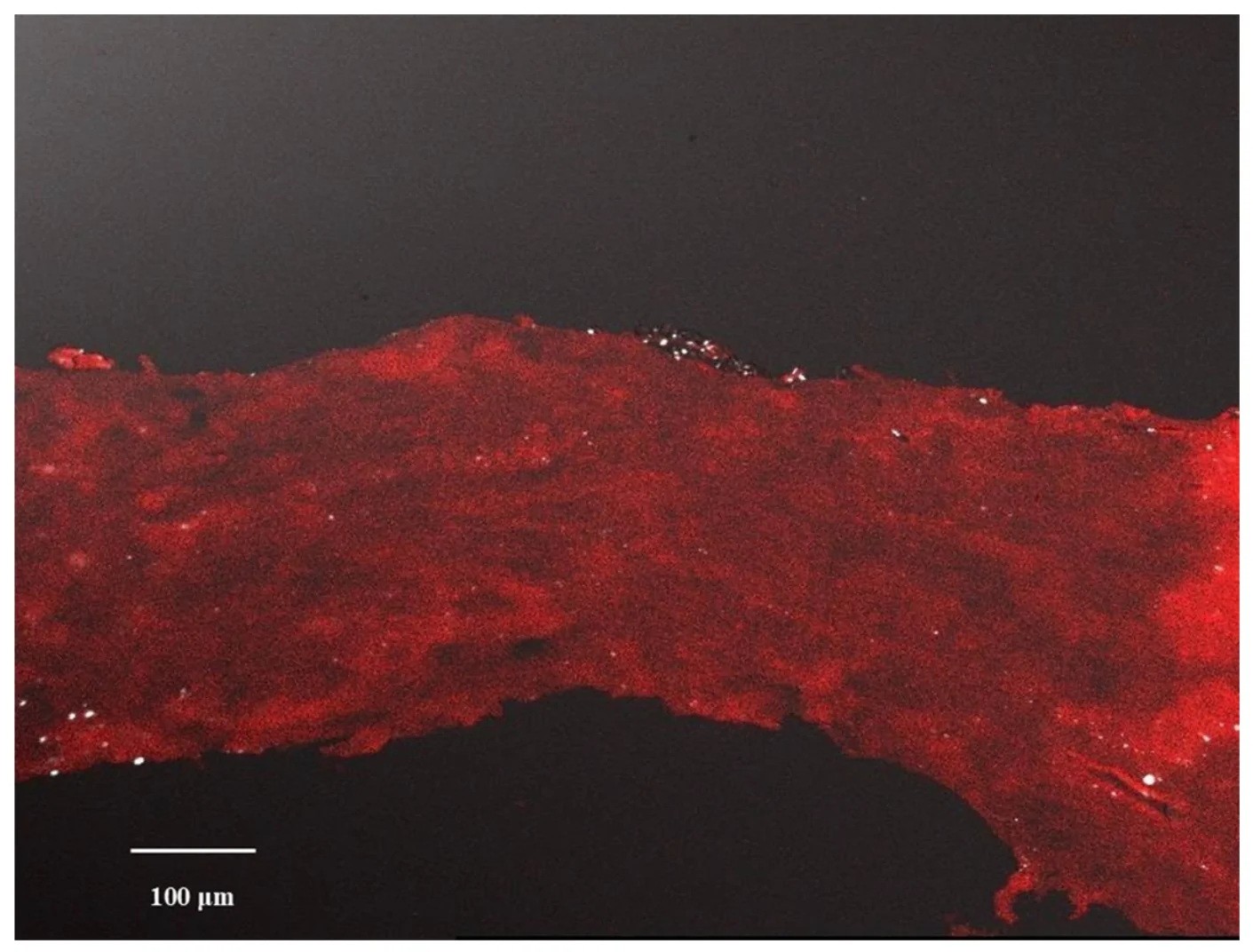
Confocal laser scanning microscopy (CLSM) image of skin treated with the optimal KPN-Ca-ASPMs (F9).

As shown in Figure 5, strong fluorescence was detected from the superficial skin layers and extended into deeper layers of the skin, indicating effective penetration of the optimal formulation beyond the skin surface. The widespread distribution of fluorescence suggests that aspasomes facilitated enhanced skin permeation and drug deposition, which may be partly attributed in the present study to the presence of cationic surfactant in the aspasomal construct, which facilitates close interaction with the negatively charged skin surface and consequently generates a local drug reservoir for a longer time at the site of application [15]. Furthermore, the small PS of aspasomal formulation could facilitate its penetration and distribution throughout skin layers [37]. Hence, the observed performance is likely influenced by the combined characteristics of the complete aspasomal formulation, including its vesicular structure, lipid composition, nanoscale size, and surface charge. These findings underscore the potential of KPN-Ca-ASPMs as an efficient vesicular carrier for improving topical drug delivery to fungal-infected skin.

#### 2.8.4. Histopathological Evaluation of Skin Tissues

**Figure 6:**
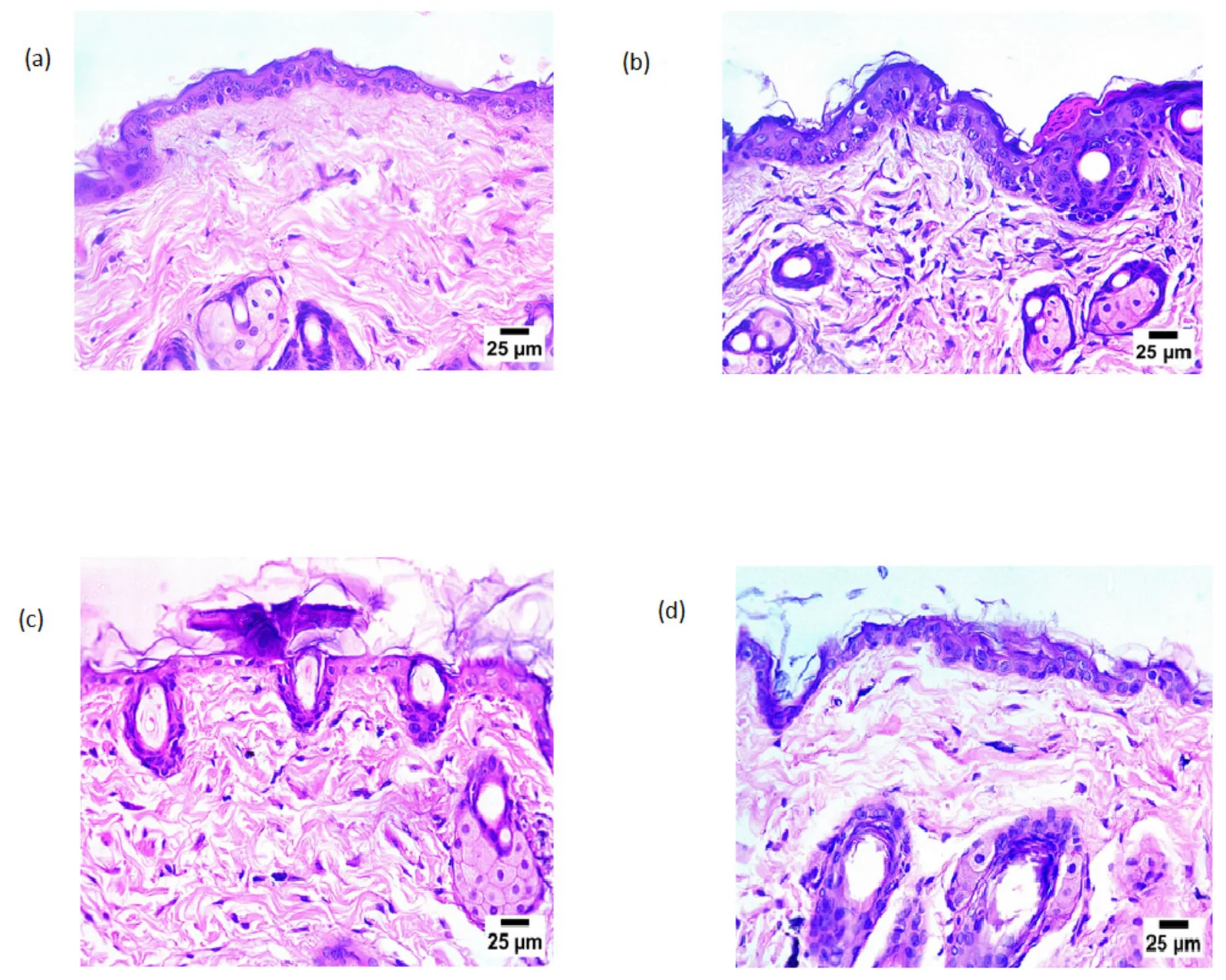
Histopathological sections of (**a**) normal control group, (**b**) positive control group, (**c**) KPN-suspension treated group, and (**d**) F9 treated group.

As shown in Figure 6, the epidermis and dermis of the normal control group exhibited a normal histological structure (a). In the positive control group, crust formation was observed on the epidermis, accompanied by infiltration of the dermis with a few lymphocytes and eosinophils (b). Considering the KPN-suspension treated group (c), photomicrographs showed the formation of a thin skin crust on the epidermis without infiltration of inflammatory cells into the dermis. In the F9 treated group (d), the epidermis and dermis retained a normal histological structure, with no marked pathological changes. Overall, the histopathological findings indicate preservation of normal skin architecture and the absence of obvious tissue damage in the F9-treated group under the tested conditions. The findings also suggest reduced inflammatory changes compared with the KPN-suspension treated group.

## 3. Materials and Methods

### 3.1. Materials

Ketoprofen (KPN) was a kind gift from the Egyptian International Pharmaceutical Industries (EIPICO; Cairo, Egypt). Ascorbyl palmitate, phosphatidyl choline (PC), cetyltrimethylammonium bromide (CTAB), dimethyldidodecylammonium bromide (DDAB), and dialysis membrane (molecular weight cut-off of 12,000–14,000 g/mol) were purchased from Sigma Aldrich (Darmstadt, Germany). Ethanol and chloroform were supplied from Merck KGaA (Darmstadt, Germany). High analytical grade was used for the remaining chemicals and reagents. The used water was laboratory-produced distilled water obtained using a laboratory water-distillation system.

### 3.2. Preparation of Ketoprofen-Cationic Aspasomes (KPN-Ca-ASPMs)

KPN-cationic aspasomes (KPN-Ca-ASPMs) were fabricated by the modified ethanol injection method using various amounts of ascorbyl palmitate and different types of cationic SAA. First, PC (100 mg) with ascorbyl palmitate, KPN (500 mg) and cationic SAA were weighed and dissolved in 2 mL of a 1:1 *v*/*v* ethanol and chloroform mixture. Different amounts of ethanol were dissolved in the distilled water and then introduced into the lipophilic mixture. A magnetic stirrer (Model MSH-20D, GmbH, Berlin, Germany) was used to completely evaporate the solvent at a temperature of 25 °C at 1500 rpm for 30 min. After that, the dispersions were kept at 4–8 °C.

### 3.3. Statistical Design and Factorial Analysis

D-optimal design was used for statistical optimization of KPN-Ca-ASPMs, ensuring efficient data collection with minimal experimental runs [28]. The experimental design was generated using Design Expert^®^ software (Version 13, Stat-Ease, Inc., Minneapolis, MN, USA), which can also establish mathematical correlations between input and output variables and assess the importance of the selected factors [38]. For a three-factor D-optimal design, a total of 15 experiments were run. The study design aimed to assess the influence of 3 independent variables, namely, ascorbyl palmitate amount (X_1_), ethanol concentration (X_2_), and cationic SAA type (X_3_). EE% (Y_1_), PS (Y_2_), and ZP (Y_3_) were set as dependent variables. Table 4 shows the examined variables, their levels, and the goal constraints. The design matrix, the components of the developed formulations, and their complete characterization are shown in Table 4.

### 3.4. In Vitro Evaluation of the Prepared KPN-Ca-ASPMs

#### 3.4.1. Determination of Entrapment Efficiency (EE%)

The direct method was utilized for the determination of the EE% of KPN within aspasomes. Briefly, 1 mL of aspasomes dispersion was subjected to high-speed centrifugation for 1 h at 20.000 rpm and 4 °C (Sigma-3K30, Sigma Laborzentrifugen GmbH, Osterode am Harz, Germany). Then, the sedimented vesicles were lysed with methanol and spectrophotometrically analyzed at λ_max_ = 257 nm [7]. The mean ± standard deviation (SD) was used to express the findings of the duplicate measurements. The EE% was determined using the following equation [39]:

(1) $$EE\%=\frac{Entrapped\;KPN\;amount}{Total\;KPN\;amount}\times 100$$

#### 3.4.2. Determination of Particle Size (PS), Polydispersity Index (PDI), and Zeta Potential (ZP)

Key metrics, including particle size (PS), polydispersity index (PDI), and zeta potential (ZP), were analyzed, as they are crucial markers for the electrostatic stability and homogeneity of the nanosystem [40]. Following proper dilution, PS, PDI, and ZP of KPN-Ca-ASPMs were measured using a Zetasizer (Malvern Instruments Ltd., Worcestershire, UK). The instrument measures the electrophoretic mobility of the dispersed particles, which is subsequently converted to zeta potential using the Henry equation with the Smoluchowski approximation. All measurements were performed in triplicate. The mean ± standard deviation (SD) was used to express the results [41].

#### 3.4.3. Statistical Optimization of the Prepared KPN-Ca-ASPMs

In alignment with QbD principles, the optimal formulation is derived from the multidimensional combination of the input variables to satisfy our critical quality attributes (CQAs) [42]. This systematic approach ensures that the repurposed KPN nanoparticles maintain structural integrity and therapeutic efficacy within a robust design space [20]. The optimization process utilized a desirability function that prioritized maximum EE% and ZP while simultaneously minimizing PS. Based on the analysis of variance (ANOVA) and the integration of response surface methodology, the formulation with the highest desirability index was identified as the optimal KPN-Ca-ASPMs, which was then subjected to confirmatory characterization and microbiological evaluation to validate the model’s predictive power.

### 3.5. In Vitro Drug Release

The release of KPN from its aqueous suspension and the optimal KPN-Ca-ASPM formulation was performed using the dialysis bag diffusion technique in a horizontal shaking water bath (Maxturdy-30, Witeg Labortechnik Gmbh, Wertheim, Germany) [7]. A cellulose membrane (molecular weight cut-off: 12,000–14,000 Da) acted as a controllable diffusion barrier layer that separated the receptor and donor compartment. The latter was loaded with 0.5 mL of the optimal KPN-Ca-ASPMs. Fifty milliliters of phosphate buffer (pH 5.5) was kept in the receptor compartment which was continuously stirred (150 rpm) to ensure even mixing and maintained at 37 ± 0.05 °C to mimic physiological conditions [8]. At predetermined time intervals (1, 2, 3, 4, 5, and 6 h), 3 mL samples were taken out from the receptor compartment and promptly replenished with an equal volume of fresh phosphate buffer to maintain the sink condition [43]. In each sample, the concentration of KPN was quantitatively measured spectrophotometrically at *λ*_max_ = 257 nm. Release data are shown as mean ± SD of triplicate measurements. ANOVA was used to perform a comparative statistical analysis of release profiles; a *p*-value of ˂0.05 was considered statistically significant.

### 3.6. Transmission Electron Microscopy

A transmission electron microscope (TEM) (JEM-1230, JEOL Ltd., Tokyo, Japan) was used to analyze the morphological features of the optimal KPN-Ca-ASPM formulation. After applying a little drop of the formulation to a carbon coated copper grid, the grid was allowed to dry and inspected under a TEM for imaging [44].

### 3.7. Differential Scanning Calorimetry (DSC)

After being frozen, the optimal KPN-Ca-ASPM was lyophilized for a whole day at −45 °C and 7 × 10^−2^ mbar pressure using a lyophilizer (Novalyphe-NL 500, Savant Instruments, New York, NY, USA). A previously calibrated differential scanning calorimeter (Shimadzu DSC 50; Kyoto, Japan) was used to record the thermograms of MFC and lyophilized optimal aspasomes. In brief, 3–4 mg samples of each were heated in standard aluminum pan over a temperature range of 10 °C to 300 °C at a constant scanning rate of 10 °C/min under an inert nitrogen flow (30 mL/min) [45].

### 3.8. Effect of Short-Term Storage Stability

To evaluate the physical and functional stability of aspasomes formulation, the optimal KPN-Ca-ASPM formulation was refrigerated for 3 months in sealed amber glass. Periodically, the samples were examined for any indications of particle growth or phase separation. After completing the storage period, EE%, PS, PDI, and ZP were re-measured and compared to the fresh samples. After that, SPSS^®^ software version 22.0 (SPSS Inc., Chicago, IL, USA) was used to statistically analyze the data using a paired *t*-test [46].

### 3.9. In Vitro Antimicrobial Activity

#### 3.9.1. Determination of the Minimum Inhibitory Concentration (MIC)

The antifungal activity of KPN against *Candida albicans* ATCC10231 was tested [47]. As previously mentioned and in accordance with the Clinical and Laboratory Standards Institute’s criteria, the microdilution method was used to calculate the minimum inhibitory concentration (MIC) [48,49]. In brief, a range of concentrations (12.5–0.0224 mg/mL) was obtained by serially diluting the tested KPN formulations in Sabouraud dextrose broth (SDB). Each well of sterilized 96-well microtiter plates received a standardized fungal inoculum (105–106 CFU/mL). Fungal growth was evaluated visually and spectrophotometrically by measuring the optical density at 600 nm using a microplate reader (Biotek Synergy 2, SLFA model, Winooski, VT, USA) after the microplates had been incubated at 30 °C for 24 to 48 h. The lowest concentration with no visible fungal growth was found to be MIC. The experiment was conducted three times independently, and the mean ± standard deviation was used to report the results.

#### 3.9.2. Microbiological and In Vivo Analysis

##### Animals

Healthy adult male mice were employed for the in vivo evaluation study. The animals were housed in sanitized, well-ventilated cages under controlled laboratory conditions (25 ± 2 °C) with a 12 h light/dark cycle. Throughout the trial, a well-balanced commercial diet and unlimited access to clean drinking water were offered. All animals underwent thorough veterinarian screening before initiating the experiment in order to verify their general health and rule out any signs of inflammatory abnormalities [50]. All the animal procedures were approved by the Research Ethics Committee of the Faculty of Pharmacy, Cairo University (Approval# MI 4107) following the “Guide for the Care and Use of Laboratory Animals” published by the Institute of Laboratory Animal Research (Washington, DC, USA).

##### In Vivo Fungal Skin Infection Model

The optimal KPN-Ca-ASPM was tested in vivo to validate its antifungal activity in a skin infection model as reported before with slight modifications [8,51]. Eighteen BALB/C male mice (7 weeks old) were used in the fungal skin infection model. Before the experiment, the dorsal backs of the mice were shaved. The lower backs of the mice were injected intradermally with 100 µL *Candida albicans* ATCC10231 suspended in sterile phosphate-buffered saline (PBS) (9 × 10^9^ CFU). Mice were randomly distributed into three groups (six mice per group, *n* = 6). At 48 h post-infection, the first group was treated topically with the optimal KPN-Ca-ASPMs (25 mg/mL). The second group was treated with KPN-suspension (25 mg/mL). The third group was used as the negative control (did not receive any treatment). All groups were treated at the site of infection by topical application of 100 µL of the treatment using a soft plastic tip once daily for three days. After 24 h from the last treatment, the experiment was terminated and the animals were euthanized; the skin lesion was removed, then homogenized using 0.5 mL saline (homogenizer, DAIHAN-scientific-pacificlab) [52,53]. Samples were diluted 10 fold in PBS and tested for aerobic viable count by plating on Sabouraud dextrose agar followed by incubation at 30 °C. After 48 h incubation, the plates were inspected for colony-forming units (CFU) and the results of the tested groups were analysed and compared.

##### In Vivo Skin Permeation Study by Confocal Laser Scanning Microscopy (CLSM)

The skin distribution of the aspasomal formulation was evaluated using a fluorescein diacetate (FDA), lipophilic fluorescent probe [54]. The optimal KPN-Ca-ASPM formulation was prepared as previously mentioned, but 10 mg of FDA was used in place of KPN [8]. In this experiment, male albino Wistar rats were employed. Before the experiment, the rats were anesthetized using a ketamine and xylazine mixture [55]. The dorsal side of each was then shaved, disinfected, and examined for any cracks. The shaved portion of the rat skin (2.5 cm diameter) was treated with the FDA-loaded KPN-Ca-ASPM formulation. The animals were sacrificed after six hours. A microtome (Cambridge Instruments Ltd., Cambridge, UK) was used to obtain longitudinal slices of the treated skin following the application period. After that, the tissue sections were examined by CLSM (LSM 710; Carl Zeiss, Jena, Germany). The captured confocal images were analyzed using LSM Image Browser (release 4.2; Carl Zeiss MicroImaging GmbH, Jena, Germany).

##### Safety Assessment via Histopathological Evaluation

Following the last treatment, skin samples from the infection sites of all experimental groups (normal control, positive control—infected group, not treated—KPN-suspension, and optimal KPN-Ca-ASPM) were gathered and promptly fixed in 10% neutral buffered formalin for 24 h. After that, the fixed tissues were cut, cleaned under running water, clarified in xylene, dehydrated in ethyl alcohol, and embedded in paraffin wax. In accordance with the procedure outlined by Bancroft et al., thin slices (4–6 µm) were produced using a rotary microtome, mounted on glass slides, and stained with hematoxylin and eosin (H&E) [56]. A light microscope (Leica Microsystems, Wetzlar, Germany) was used to view the stained sections in order to evaluate the overall histopathological examination.

## 4. Conclusions

This study successfully demonstrated the rational development of Quality by Design-optimal ketoprofen-loaded cationic aspasomes (QbD-KPN-Ca-ASPM) as a novel topical nanoplatform for the treatment of cutaneous candidiasis. The formulation was prepared by the ethanol injection technique and systematically optimized within a QbD framework using a D-optimal experimental design to achieve predefined critical quality attributes, including high entrapment efficiency, nanosized vesicles, and a high positive zeta potential. The optimal KPN-Ca-ASPM exhibited enhanced drug release, satisfactory physicochemical short-term stability, and enhanced skin distribution, which translated into significantly improved antifungal efficacy in a murine model of cutaneous candidiasis compared with free KPN. Histopathological evaluation further confirmed the restoration of skin integrity without detectable tissue damage, supporting the safety of the developed formulation. Collectively, these findings provide preliminary preclinical evidence supporting the repurposing of KPN as a topical antifungal agent and demonstrate that QbD-guided cationic aspasomes represent a robust platform for enhancing cutaneous drug delivery and therapeutic performance. Nevertheless, translation of these findings to human cutaneous candidiasis requires further investigation, including longer-term toxicity, pharmacokinetic, and efficacy studies in more clinically representative models.

## Figures and Tables

**Table 1:** D-optimal design for optimization of ketoprofen-cationic aspasomes.

Response	R^2^	Adjusted R^2^	Predicted R^2^	Adequate Precision	Significant Factors
EE%	0.94	0.92	0.88	23.38	X_1_, X_2_, X_3_
PS	1	0.99	0.99	63.15	X_1_, X_2_, X_3_
ZP	0.96	0.94	0.87	27.48	X_1_, X_3_

Abbreviations: EE%, entrapment efficiency percent; PS, particle size; ZP, zeta potential.

**Table 2:** Composition and characterization of the prepared KPN-Ca-ASPMs.

Formulation Code	Ascorbyl Palmitate Amount (mg)	Ethanol Concentration (*v*/*v*)	Type of Cationic SAA	EE%	PS (nm)	PDI	ZP (mV)
F1	15	7.5	CTAB	79.95 ± 0.71	199.62 ± 0.70	0.310 ± 0.13	28.99 ± 0.20
F2	20	10	CTAB	76.97 ± 0.52	88.75 ± 3.65	0.243 ± 0.041	29.50 ± 0.55
F3	10	10	CTAB	81.43 ± 7.11	66.38 ± 9.87	0.445 ± 0.048	36.00 ± 0.20
F4	10	10	CTAB	78.19 ± 6.89	72.40 ± 8.66	0.445 ± 0.038	36.00 ± 0.20
F5	20	5	CTAB	78.00 ± 4.00	417.13 ± 0.15	0.232 ± 0.034	13.40 ± 0.60
F6	20	10	CTAB	75.00 ± 1.14	73.00 ± 2.00	0.325 ± 0.002	33.29 ± 0.68
F7	15	5	CTAB	84.21 ± 4.65	298.18 ± 7.56	0.312 ± 0.038	24.63 ± 0.34
F8	10	5	CTAB	85.33 ± 6.96	180.88 ± 0.45	0.390 ± 0.066	39.90 ± 0.31
F9	10	5	DDAB	91.30 ± 10.01	213.24 ± 2.20	0.324 ± 0.068	51.70 ± 0.65
F10	20	5	DDAB	84.38 ± 0.08	104.46 ± 2.89	0.450 ± 0.003	32.80 ± 1.40
F11	20	10	DDAB	79.81 ± 3.65	89.00 ± 4.89	0.306 ± 0.003	38.44 ± 2.67
F12	15	5	DDAB	87.66 ± 0.59	168.04 ± 5.60	0.290 ± 0.057	41.60 ± 0.35
F13	17.5	7.5	DDAB	83.74 ± 0.487	138.87 ± 2.87	0.310 ± 0.34	40.60 ± 1.67
F14	20	10	DDAB	79.09 ± 4.65	90.00 ± 2.56	0.312 ± 0.002	36.11 ± 2.76
F15	10	7.5	DDAB	89.19 ± 1.10	313.71 ± 2.19	0.440 ± 0.001	38.88 ± 0.28

Data are presented as mean ± SD. Abbreviations: KPN-Ca-ASPMs, ketoprofen-cationic elastic aspasomes; SAA, surface active agent; EE%, entrapment efficiency percent; PS, particle size; PDI, polydispersity index; ZP, zeta potential.

**Table 3:** Predicted and observed values for the optimal KPN-Ca-ASPM (F9).

Response	Y_1_: EE%	Y_2_: PS (nm)	Y_3_: ZP (mV)
Observed values	91.30	213.24	51.70
Predicted values	90.72	214.00	50.47

Abbreviations: EE%, entrapment efficiency percent; PS, particle size; ZP, zeta potential.

**Table 4:** D-optimal design for optimization of KPN-Ca-ASPMs.

Factors (Independent Variables)	Levels	
	Low (−1)	High (+1)
X_1_: Ascorbyl palmitate amount (mg)	10	20
X_2_: Ethanol concentration (*v*/*v*)	5	10
X_3_: Cationic SAA type	DDAB	CTAB
Responses (dependent variables)	Constraints	
Y_1_: EE (%)	Maximize	
Y_2_: PS (nm)	Minimize	
Y_3_: ZP (mV)	Maximize (as absolute value)	

Abbreviations: EE%; Entrapment Efficiency Percent, PS; Particle Size, ZP; Zeta Potential.

## Data Availability

The data generated and/or analyzed during the current study are available from the corresponding author upon reasonable request.

## Abbreviations

The following abbreviations are used in this manuscript:

EE%	Entrapment efficiency
PS	Particle size
PDI	Polydispersity index
ZP	Zeta potential
KPN	Ketoprofen
ASPM	Aspasomes
QbD	Quality by Design
CQAs	Critical quality attributes
